# Supplementary material for: Mass-Synthesized Solution-Processable Polyimide Gate Dielectrics for Electrically Stable Operating OFETs and Integrated Circuits
Source: Polymers (Basel). 2021 Oct 28;13(21):3715. doi: 10.3390/polym13213715 (PMC8586921; doi:10.3390/polym13213715)
Supplement: Supplementary file 1 [file polymers-13-03715-s001.zip › polymers-1417415-supplementary.pdf]

Supporting Information

Mass Synthesized Solution Processable Polyimide Gate Dielectrics for Electrically Stable Operating OFETs and Integrated Circuits

Rixuan Wang [1,†], Joonjung Lee [2,†], Jisu Hong [3,4,†], Hyeok-jin Kwon [4], Heqing Ye [1], Juehyun Park [5], Chan Eon Park [4], Joon Ho Kim [1,2], Hyun Ho Choi [3,6,*], Gyuyoung Um [5,*] and Se Hyun Kim [1,2,*]

[1] School of Chemical Engineering, Yeungnam University, Gyeongsan 38541, Republic of Korea; nl910213@gmail.com (R.W.); yeheqing5420@gmail.com (H.Y.); joon@ynu.ac.kr (J.H.K.)
[2] Department of Advanced Organic Materials, Yeungnam University, Gyeongsan 38541, Republic of Korea; joon@utp.or.kr
[3] Research Institute for Green Energy Convergence Techonology, Gyeongsang National University, Jinju 52828, Republic of Korea; jisu225@postech.ac.kr
[4] Department of Chemical Engineering, Pohang University of Science and Technology, Pohang 37673, Republic of Korea; hj1370@postech.ac.kr (H.-j.K.); cep@postech.ac.kr (C.E.P.)
[5] COMEC Corporation, Pyeongtaek 17957, Republic of Korea; komec66@daum.net
[6] Department of Materials Engineering and Convergence Technology, Gyeongsang National University, Jinju 52828, Republic of Korea
* Correspondence: hh.choi@gnu.ac.kr (H.H.C.); eky100@hanmail.net (G.U.); shkim97@yu.ac.kr (S.H.K.); Tel.: +82-53-810-2788 (S.H.K.)
† These authors contributed equally to this work.

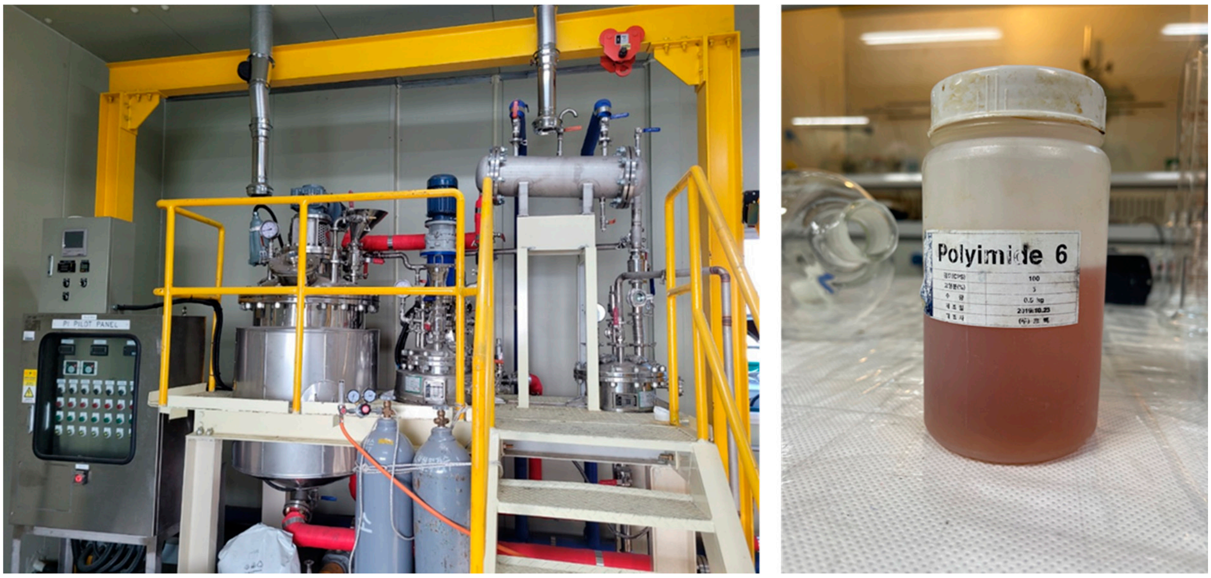

Figure S1. Industrialized reactor photograph and prepared PAA solution in 1L plastic bial.

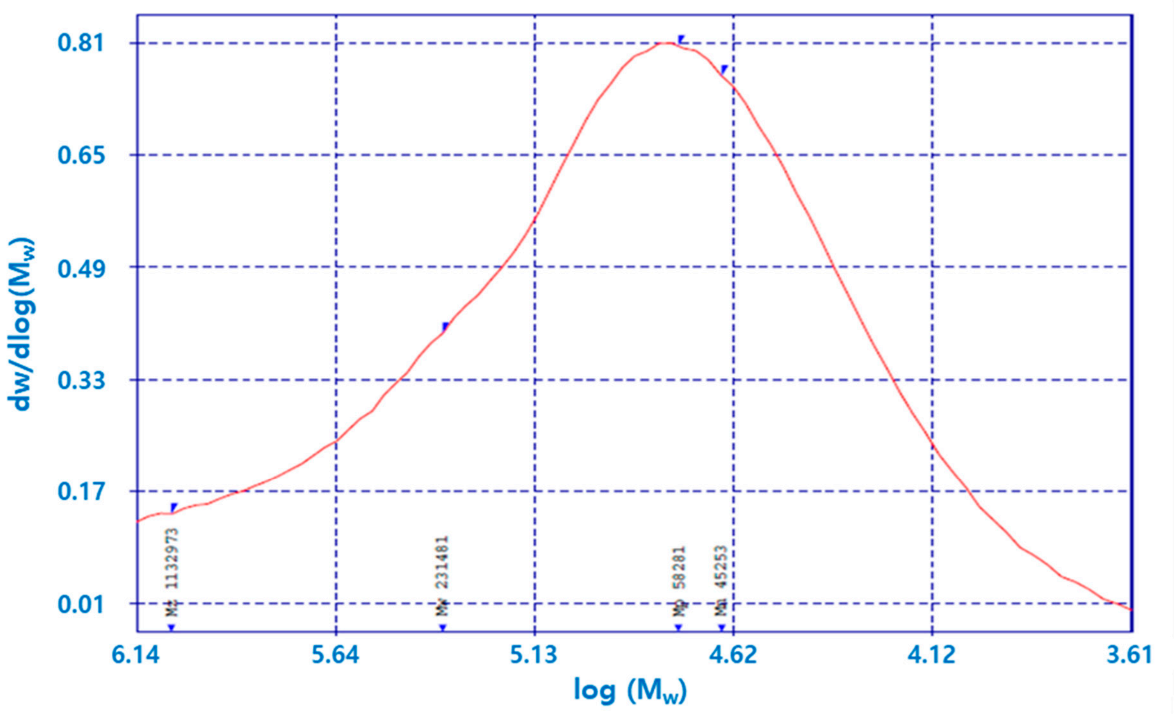

Figure S2. GPC graph of prepared PAA solution.

Table S1. Molecular weight of PAA solution

**Molecular Weight**	**M_n**	**M_p**	**M_w**	**Mz**	**M_z+1**	**PD**
**PAA Solution**	**45253**	**58281**	**231481**	**1132973**	**2113610**	**5.11517**
